# Assessing the composition of nurses in South Africa versus the WHO perspective

**DOI:** 10.4102/curationis.v48i1.2762

**Published:** 2025-10-24

**Authors:** Fatima Abrahams, Sanjana Brijball Parumasur

**Affiliations:** 1 Discipline of Human Resource Management and Industrial Relations, School of Commerce, University of KwaZulu-Natal (Westville Campus), Durban, South Africa

**Keywords:** healthcare service delivery, nurses-to-population ratio, shortage of healthcare workers, South Africa, World Health Organization

## Abstract

**Background:**

The shortage of healthcare workers is a global phenomenon with many regions experiencing difficulties in providing a healthcare workforce to meet their demand for healthcare. This situation has led to developed countries recruiting staff from developing countries, thereby causing a shortage in the source country, referred to as a brain drain. Given the negative impact of the turnover of healthcare staff on patient safety, service delivery, healthcare cost and staff morale within healthcare facilities, this research study investigates the current composition of nurses in South Africa versus the World Health Organization (WHO) standard.

**Objectives:**

The study aims to enhance the retention of healthcare workers.

**Method:**

Secondary data collection was utilised to assess how the composition of nurses in South Africa compares against the WHO standard. Non-probability sampling was adopted to draw the study sample to collect data in the original study from nurses registered with the South African Nursing Council (SANC), employing a mixed-method approach to data collection and analysis.

**Results:**

The results indicate that South Africa’s nurses-to-population ratio exceeds the WHO standard.

**Conclusion:**

Although South Africa’s nurse-to-population ratio exceeds the WHO standard, these figures do not accurately reflect the nursing situation in the country.

**Contribution:**

Recommendations are presented which, when effectively implemented, have the potential to enhance the retention of professional nurses and improve healthcare service delivery in South Africa.

## Introduction

For every nation, having a reliable and effective healthcare system is crucial. The expansion of knowledge and service-based economy, globalisation and the liberalisation of trade in services have all contributed to an increase in the need for skilled workers. Such workers are moving around more as our societies grow increasingly interdependent and linked. Similar to other labour markets, the health sector has a high rate of professional mobility, which has a negative impact on nations, and South Africa is no exception. One of the major issues facing the South African healthcare structure is the increasing scarcity of qualified medical personnel, especially nurses. Nurses, as the backbone of the healthcare in this country, play a significant role in patient care, yet their numbers are not keeping pace with the country’s population growth. Nurses may be leaving the country altogether because the rate of nurse output does not seem to be translating into higher ratios. According to Pillay (2009), a sizeable portion of nurses depart South Africa each year.

The Health Department recently released a Draft (HR) Strategy for the health sector to be achieved by 2030, which identifies the need for a plan to address human capital issues as part of addressing the deteriorating South African healthcare system. The HR strategy document highlights a significant decrease in the composition of South African healthcare professionals, including nurses, thereby necessitating an investigation of the nurse-to-population ratio in South Africa. Because of the increased strain on the remaining nurses, nurses who leave may experience a decline in morale and productivity (Mealer & Moss 2016). This can lead to further work dissatisfaction and an increase in nurse turnover (Bae 2022). The efficacy and efficiency of healthcare organisations are impacted by the loss of experienced nurses, which also leads to a decline in organisational competence, a lack of continuity and the loss of institutional memory (Hayward et al. 2016).

South Africa has a deteriorating healthcare system, which is increasingly unable to meet the healthcare demands of the country. The lack of data and inconsistent data for healthcare workers, including internal migration and emigration, leads to an inability to do workforce planning and, therefore, ineffective utilisation of existing resources. In addition, the lack of tracking of healthcare professionals in the country also leads to an inability to assess the current migration situation among healthcare professionals, including nurses. It was found that the ratio decreased by 9.6% to 47 nurses per 10 000 population, which is still in excess of the WHO standard (South African Nursing Council [SANC] 2021). Given the problems experienced in the country’s healthcare sector, an exploration of this ratio is required. However, the high percentage of registered nurses who do not practice nursing or have taken on administrative positions is not taken into consideration, despite South Africa’s nurse-to-population ratio is higher than that of the World Health Organization (WHO). This investigation will assess present trends, projected shortages and potential strategies to improve nurse recruitment, training and retention – key factors in revitalising South Africa’s deteriorating healthcare system.

### Objectives of the study

#### Main objective

- To assess how the composition of nurses in South Africa compares against the WHO standard.

#### Specific objective

- To describe the attrition rate of healthcare professionals in South Africa.

### Literature review

A Healthcare Profile for South Africa, developedat the beginning of 2020, using data between 2016 and 2018, indicates that the WHO’s standard for nurses-to-population ratio is 25 per 10 000 population (Foundation for Innovative New Diagnostics [FIND] 2020). Based on this report, South Africa currently sits above the WHO recommendation at 52 nurses per 10 000 population. A 2020 report published by Statistics South Africa (Stats SA) indicates that the country’s population is thought to have grown to 59.62 million people. To confirm the aforementioned nurses-to-population ratio for South Africa, the population size was compared to the total number of nursing registrations at the end of 2020.

Nursing professionals have been identified as a critical human resource in the healthcare system of South Africa, as they constitute 80% of the total healthcare workforce in South Africa (National Department of Health [NDOH] 2011). It is therefore clear that a high turnover of nurses has a direct impact on healthcare service delivery. The majority of nurses move for financial incentives as they are unable to make a living with the salaries offered in the South African healthcare sector. However, upon achieving their financial objectives, most of them returned to South Africa (Pretorius 2018). George, Atujuna and Gow (2013) mentioned that financial and incentives influence the migration of healthcare workers and concluded that the Occupation Specific Dispensation, which was implemented in the public health sector in 2007, enhanced the state’s capacity to recruit and retain experienced staff through improved compensation. Furthermore, opportunities for growth and development must be created, and nurses’ working conditions as well as nursing accommodation should be improved. Management support must be offered, and the recruitment policy must be reviewed to avoid nepotism and political appointments (Chiloane 2017).

A study carried out by WHO in 2015 found that data available for the healthcare workforce in South Africa do not provide good insight into the internal and external movement of the healthcare workforce in the country (WHO 2015). The SANC maintains registration information for nurses; however, those records are unable to distinguish between nurses actively practising from those in retirement or working overseas (WHO 2017). This study seeks to enhance the retention of professional nurses in the South African healthcare facilities by assessing the composition of nurses in South Africa against the WHO norm.

The study is underpinned by Hertzberg’s two-factor theory, where the motivation and hygiene factors influence nurses’ productivity. In an environment often defined by work overload and poor working conditions, focusing on both hygiene factors (remuneration and working conditions) and motivators (like recognition and advancement opportunities) can trigger nurses’ satisfaction and the inner drive to perform well.

## Research methods and design

The method of data collection has been confined to desktop research or secondary data. Both quantitative and qualitative data were collected and analysed, and all sources of statistics and databases are outlined and duly referenced. Secondary data were extracted from research studies, which collected primary data from professional nurses in South Africa and from reports containing data collected in connection with the country’s healthcare system and workforce. The study adopted a descriptive and analytical design rooted in qualitative and quantitative secondary data analysis. This approach allowed for the extraction of data from pre-existing documents, databases and academic studies to identify patterns, draw comparisons and derive conclusions relevant to the state of South Africa’s nursing workforce.

South Africa’s nurses-to-population ratio needs to be investigated in order to determine how it compares with the WHO standard. Once this is determined, an exploration of the composition of the nursing workforce in South Africa can be conducted.

### Sampling technique and description of the sample

Non-probability sampling was used in the original studies to collect data from nurses registered with SANC. The researchers attempted to find the most relevant, recent and reliable information that could be generalised to the entire professional nursing workforce.

### Data collection

This research study used quantitative and qualitative secondary data collected from primary, secondary and tertiary sources. Primary sources of data for this study were collected from the WHO, the FIND, Stats SA, the SANC, the World Bank and the South African national and provincial departments of health. These organisations, statutory bodies and government departments were selected as sources for primary data because they are considered authoritative for the type of information obtained from them. In addition, secondary data sources in this study consisted of scholarly literature, including journal articles and books related to workforce planning, health systems management and retention theory. Finally, the study utilised tertiary sources, including dissertations and theses from South African universities that conducted empirical studies involving nurses. The relevant primary sources of information were searched for on Google and retrieved via official government websites, statutory body websites and organisational websites, which are considered authorities in the field of health. The search words that were entered into Google Scholar are:

- ‘professional nurses’, ‘brain drain’, ‘migration mitigation’, ‘South African hospital retention strategies’ – 38 results.
- ‘pull factors’ ‘professional nurses’ ‘brain drain migration South Africa’ – 59 results.

Examples of the primary, secondary and tertiary sources of data collection are reflected in Table 1, Table 2a and Table 2b.

**TABLE 1:** Primary sources of data collection.

Source description	Data collected	Reference	Authenticity
Foundation for Innovative New Diagnostics (FIND)	Provides SA nurses-to-population ratio vs WHO standard. Healthcare Profile for South Africa (SA) 2020	Foundation for Innovative New Diagnostics (2020)	Record composed by FIND. It is an international health NGO that originated in 2003 at the World Health Assembly in Geneva. It is an initiative for the promotion of innovative diagnostic tests for poverty-related illnesses
Statistics South Africa	Provides SA population number for as at July 2020 – SA Mid–-Year Population Growth – July 2020	Statistics South Africa (2020)	Statistics was compiled by Stats SA, which is the official government statistical service of SA. Its purpose is to produce current, reliable and official statistics, to advance economic growth, development and equality. Stats SA produces official demographic, financial and social statistics and surveys
South African Nursing Council (SANC)	Provides the age distribution of the SA nursing workforce – Nurses Age Distribution Report 2021 Report	South African Nursing Council (2022a)	Report compiled by SANC, which is the statutory body authorised to establish and control standards of nursing education and practice in SA. Data were collected in 2020 for a 2021 age analysis report. Data are collected from the nurse’s registration information received by SANC. SANC is the authority on this type of information in SA
SANC	Provides the number of nurses registered in SA and the skill level for all nurses in SA – SA Nursing Council Growth in the Registers 2021 Report	South African Nursing Council (2022a, 2022b)	The report was compiled by SANC, which is the statutory body empowered to establish and control standards of nursing training and practice in SA

Note: Please see full reference list of this article: Abrahams, F. & Parumasur, S.B., 2025, ‘Assessing the composition of nurses in South Africa versus the WHO perspective’, *Curationis* 48(1), a2762. https://doi.org/10.4102/curationis.v48i1.2762 for more information.

**TABLE 2a:** Secondary sources of data collection.

Reference	Data collected	Book	Authenticity
Coetzee, Potgieter and Ferreira (2018)	Explaining the turnover intent of nurses through the Job Demands Resource Model	Psychology of Retention: Theory, Research and Practice. Switzerland: Springer Nature	The book discusses current research and theory that guide retention strategies in the volatile, uncertain, complex and ambiguous (VUCA) environment of today. Rich information regarding the psychology of retention as it appears in actual situations worldwide may be found in several chapters

Note: Please see full reference list of this article: Abrahams & Parumasur, S.B., 2025, ‘Assessing the composition of nurses in South Africa versus the WHO perspective’, *Curationis* 48(1), a2762. https://doi.org/10.4102/curationis.v48i1.2762 for more information.

**TABLE 2b:** Tertiary sources of data collection.

Source description	Data collected	Reference	Authenticity
Regional Network for Equity in Health in East and Southern Africa (Equinet)	Data collected – The uneven distribution of healthcare workers between urban and rural areas.	Malema and Muthelo (2018)	Equinet is a regional network on equity in health in East and Southern Africa. It is a network of specialists, public community members, policymakers, government officials and others in the region who have gathered as an equity catalyst to promote and achieve shared values of fairness and social justice in health
*Curationis*?	Data collected – Turnover rate of nursing students and the primary reasons.	Roos et al. (2016)	Curation is an academic research journal that endeavours to provide a forum for the investigation of problems and experiences associated with, and supporting, nursing and midwifery best-practice development via research knowledge and problem-based knowledge sharing across the African region. The study aimed to elucidate the attrition rate at chosen SA academic institutions and the elements affecting undergraduate nursing students to abandon their nursing education at these schools
BMC health services research	Data collected – The degree to which financial incentives influence internal and external movements of South African healthcare workers and how the Occupation Specific Dispensation has enhanced state sector recruitment and retention of healthcare workers in SA. Migration of South African health workers: the extent to which financial considerations influence internal flows and external movements.	George, Atujuna and Gow (2013)	BMC Health Services Research is an open-access, peer-reviewed journal that considers studies on all aspects of health services research. The journal has a particular focus on eHealth, governance, health administration, health system quality and safety, healthcare distribution and access to healthcare, healthcare investment and finance, executing change and the health workforce. This study examines the reasons for migration, concentrating on the role of remuneration and perks. Healthcare experts from the state, private and non-governmental (NGO) healthcare institutions, found in selected peri-urban and city districts in KZN, SA, were surveyed regarding their current jobs and views concerning migration. The study utilised cross-sectional data obtained in 2009. A total of 694 healthcare professionals (430 in the state sector, 133 in the NGO sector and 131 in the private sector) were studied. An additional 11 health professionals were purposively chosen for one-on-one interviews. Odds ratios with 95% confidence intervals were calculated to conclude whether compensation affected HWs’ choices to leave

Note: Please see full reference list of this article: Abrahams & Parumasur, S.B., 2025, ‘Assessing the composition of nurses in South Africa versus the WHO perspective’, *Curationis* 48(1), a2762. https://doi.org/10.4102/curationis.v48i1.2762 for more information.

† , *Curationis* is the official journal of the Democratic Nursing Organisation of South Africa.

### Data analysis

Descriptive statistics such as frequencies, measures of central tendency and dispersion were used to present descriptive information about each of the variables in this research study to make it more coherent (Privitera 2023; Sekaran & Bougie 2016). Frequencies relate to the number of times different subcategories of a specific phenomenon transpire, from which the percentage and the cumulative percentage of its occurrence can be calculated (Sekaran & Bougie 2016). Measures of central tendency are single values inclined to be at, or close to, the middle point of a distribution (Privitera 2017). While a part of the meaning of data is always lost by reducing it to a single number, measures of central tendency guarantee that single values meaningfully represent a data set (Privitera 2017). Data will be presented using tabular representations. Data presentation includes ordering and compiling data into summary diagrammatic or optical displays.

### Trustworthiness of the data

Data validity and reliability will be ensured by collecting primary data from sources that can be considered authorities in the type of data being collected, reviewing the purpose for which the data were collected, ensuring that data are still relevant based on the period in which it was collected, only selecting data collected through scientific methods, including only data that are relevant for this research study and ensuring that the data collected are consistent with other sources (Stahl & King 2020).

### Ethical considerations

This study is based on the use of secondary data. As no primary data were collected, the study is exempt from full ethical review. An application for exemption from full ethical approval was made to the Academic Leader in the School of Management, Information Technology and Governance under the University of KwaZulu-Natal (UKZN) Ethics Research Office, and ethics consent was received on 25 July 2021. The ethics waiver number is 00012765. The UKZN Research Ethics Office issued an ethics waiver for the study because this is a desktop study and no primary data were collected at all.

## Results

### Main objective: Assess how the composition of nurses in South Africa compares against the World Health Organization standard

**FIGURE 1:**
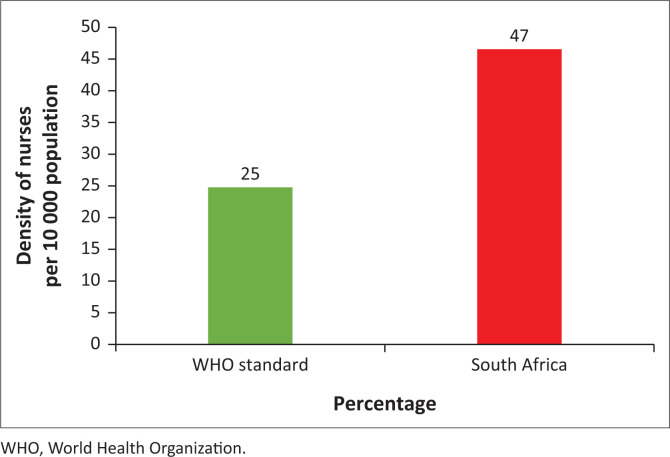
Density of nurses and midwifery (per 10 000 population).

This section provides insight into how South Africa’s nurses-to-population ratio compares to the WHO standard and outlines some of the factors which may have a negative impact on the nurses-to-population ratio in the country (Table 3 and Figure 1).

**TABLE 3:** Density of nurses and midwifery (per 10 000 population).

Constituency	Nurses per 10 000 population
World Health Organization standard	25
South Africa	47

*Source*: Data compiled from Foundation for Innovative New Diagnostics (FIND), 2020, p. 1, viewed 10 May 2021, from https://archive.finddx.org/wp-content/uploads/2020/01/5A_South-Africa_Healthcare-profile.pdf; South African Nursing Council (SANC), 2021, Age distribution, p.,1, viewed 14 April 2021, from https://www.sanc.co.za/wp-content/uploads/2022/01/Stats-2021-1-Provincial-Distribution.pdf; Statistics South Africa (StatsSA), 2020, SA mid-year population growth July 2020, p. 1, viewed 10 May 2021, from http://www.statssa.gov.za/?p=13453

Table 3 and Figure 1 indicate that WHO recommends a minimum threshold of 25 nurses and midwives per 10 000 population to ensure a country can provide essential health services and progress towards Universal Health Coverage (UHC). This percentage serves as a global benchmark and is widely used to assess whether countries have sufficient nursing personnel to meet the health demands of their populations. Furthermore, Table 3 and Figure 1 reflect that South Africa currently operates above the WHO standard for nurses-to-population ratio at 47 nurses for every 10 000 population (FIND 2020). According to this ratio, South Africa has an adequate number of nurses to deliver the required healthcare services in the country, which necessitated assessing their skill level (Table 4) and age distribution (Table 5) and skill level of nurses close to retirement age (Table 6).

**TABLE 4:** Skill level of nurses in South Africa 2021.

Title	% of nursing workforce
Registered nurses or midwives	55
Enrolled nurses or midwives	22
Nursing auxiliaries	23

*Source*: Data compiled from South African Nursing Council, 2022b, *Registrations and listed qualifications – Calendar year 2021*, p. 1–38, viewed 14 April 2021, from https://www.sanc.co.za/wp-content/uploads/2022/04/Stats-2021-2-Registrations-and-Listed-Quals.pdf.

**TABLE 5:** Percentage of South African nurses 50 years old and above.

Nurses below and above 50 years	%
Nurses below 50 years old	62
Nurses 50 years old and above	38

*Source*: Data compiled from South African Nursing Council, 2022a, *Age distribution: Registered nurses/midwives*, p. 1–7, viewed 14 April 2021, from https://www.sanc.co.za/wp-content/uploads/2022/01/Age-stats-2021.pdf

**TABLE 6:** Skill level of nurses 50 years old and above.

Title	Percentage
Registered nurses or midwives	68
Enrolled nurses or midwives	17
Nursing auxiliaries	15

*Source*: Data compiled from South African Nursing Council, 2022a, *Age distribution: Registered nurses/midwives*, p. 1 -7, viewed 14 April 2021, from https://www.sanc.co.za/wp-content/uploads/2022/01/Age-stats-2021.pdf

**FIGURE 2:**
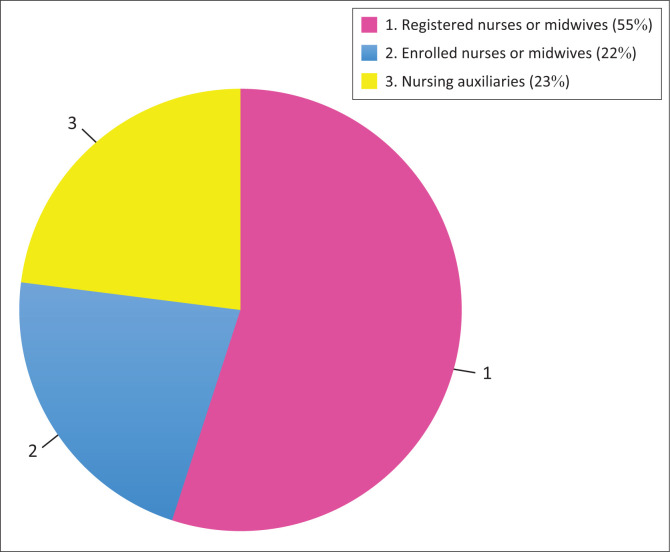
Skill level of nurses in South Africa 2021.

Table 4 and Figure 2 reflect that South Africa has mainly registered nurses or midwives (55%). This group represents the highest level of qualification among nurses in South Africa. Registered nurses (RNs) and midwives typically hold a four-year diploma or degree in nursing, with full professional registration from SANC. Furthermore, the auxiliary nurses constitute 23% of the nursing workforce. Nursing auxiliaries receive 1-year basic training and are primarily responsible for non-specialised, routine care, such as patient hygiene, feeding and basic observation. Lastly, enrolled nurses or midwives constitute 22% of the workforce, playing a key role in healthcare. Enrolled nurses and midwives typically complete a 2-year training programme and work under the supervision of registered nurses.

**FIGURE 3:**
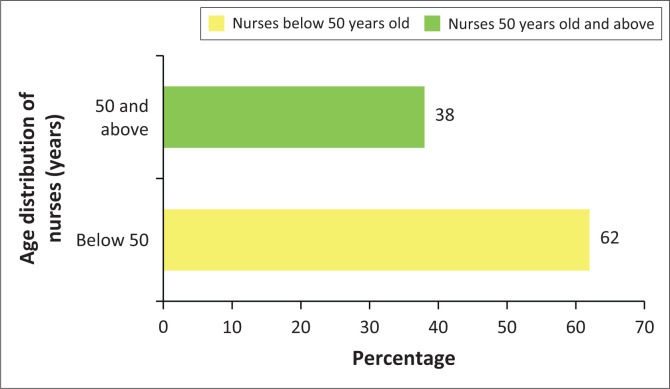
Age distribution of nurses.

Table 5 and Figure 3 show that South Africa has an ageing nursing workforce with as many as 38% of the country’s nurses who are set to retire during the next 10 to 15 years (SANC 2021). The nurses in this category also include nurses who are between the ages of 60 to 69 years, as well as those above 69 years (SANC 2021). This indicates that almost 40% of all nursing professionals in the country would have to be replaced during the next 10 to 15 years, which is a huge loss of skills and experience in the profession.

**FIGURE 4:**
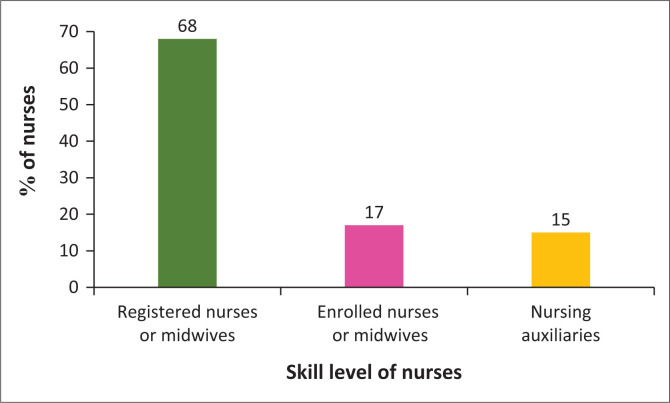
Skill level of nurses 50 years old and above.

Table 6 and Figure 4 show that there is a percentage of nurses who are above retirement age who may not be making a full contribution to the nursing workforce in the country. In particular, Table 6 and Figure 4 indicate that 68% of the 50-year-old and above nurses are registered nurses or midwives, who are the most skilled nurses in the profession (SANC 2021). This means that the majority of nurses retiring during the next 10 to 15 years are the most skilled nurses in the country. In addition, this distribution reveals that older nurses in South Africa are the most highly trained professionals in the nursing hierarchy. Registered nurses and midwives are the backbone of the healthcare system.

### Specific objective: Description of the attrition rate of healthcare professionals in South Africa

This section provides insight into the attrition of professional nurses in the South African healthcare sector. The turnover rate for the government departments depicted in Table 7 is based on the availability of relevant data.

**TABLE 7:** Annual turnover of professional nurses in South Africa.

Department	Year	Turnover rate (%)
National Department of Health (NDOH)	2018/2019	14
Gauteng Department of Health (GDOH)	2018/2019	10
Eastern Cape Department of Health (ECDOH)	2018/2019	10

*Source*: National Department of Health (NDOH) (2019, p. 77), Eastern Cape Department of Health (ECDOH) (2019, p. 226). Gauteng Department of Health (GDOH) (2019, p. 131)

**FIGURE 5:**
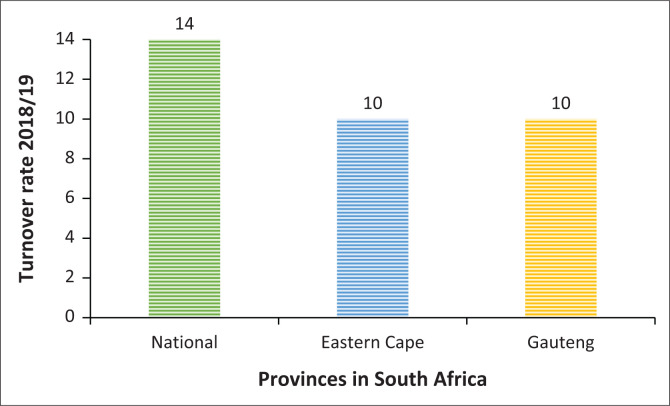
Annual turnover of professional nurses in South Africa.

Table 7 and Figure 5 depict the turnover rates recorded for NDOH and two other provinces’ health departments, including the Eastern Cape and Gauteng, for the year 2018/2019. The data displayed depicts that the NDOH has an attrition rate of 14% for professional nurses. The turnover rate recorded for professional nurses in the two provinces shown is 4% lower than the national turnover rate for this category of employees. However, given the lack of data for the rest of the provinces in South Africa, the best statistic to use in Table 7 and Figure 5 is the turnover rate recorded for the NDOH, which is 14%. This turnover rate indicates that the public healthcare sector has to replace 14% of its most qualified nurses annually.

## Discussion

### Main objective: To assess how the composition of nurses in South Africa compares against the World Health Organization standard

Good quality healthcare service provision is dependent on the workforce in the healthcare system in terms of the number and skill level of workers, how and where they work, as well as the management and deployment of the medical services, equipment and personnel (Van Rensburg 2019).

A 2017 report by the WHO revealed that although SANC maintains registration information for nurses in the country, the records do not distinguish between nurses actively practising and those in retirement or those who are working overseas (WHO 2017). Similarly, an article written by a South African trade union, Solidarity indicates that SANC does not accurately present the situation of the nursing sector in the country as the figures and statistics they use are based entirely on the nursing register and not on those who are actually working in the industry (Brits 2019). The SANC figures incorporate students, retirees and nurses who have emigrated (Brits 2019). Also, there are no organisations in South Africa where transparent and comprehensive statistics can be collected, which truly depict the situation of the nursing profession in the country (Brits 2019).

Another article published in 2019 by Solidarity contradicts the nurses-to-population ratio as it claims that one of South Africa’s greatest difficulties is staff shortages in the nursing profession. The article highlighted that the Department of Health recorded a deficit of more than 44 700 nursing personnel in 2010, and in 2016, there was a shortfall of 44 780 professional nurses while only 3595 students enrolled for the degree course. The prediction is, therefore, that the shortages will escalate (Van Rensburg 2019). South Africa’s nurses-to-population ratio is above the WHO standard, but it is not an accurate portrayal of the nursing sector in the country because of several factors, which have been identified as negatively impacting the figures. These factors include the lack of reliable data for the nursing workforce, the varying skill level of nurses, the age distribution of nurses, the burden of disease in the country and the uneven distribution of healthcare workers between the public and private healthcare sectors.

This study found that 45% of South Africa’s nursing workforce are not registered nurses (SANC 2021). This means that these workers’ contribution to the workforce is not equal to that of a registered nurse, as their scope of practice has various limitations depending on their level (*Nursing Act 33 of 2005* 2005). The nurses-to-population ratio is therefore not a true account of the nursing workforce in South Africa as the numbers are presented with the assumption that all nurses in the country are able to provide the same level of care to the people they serve. A study conducted in the United Kingdom revealed that lower registered nurse staffing and greater numbers of patients per registered nurse are linked to a greater chance of dying while in hospital (Griffiths et al. 2019). The outcome of this study emphasises the potential negative influence that shortages of registered nurses have on patient safety. The study also found that it is improbable that the negative impact of a deficit in registered nurses can be solved by improving the numbers of less qualified nursing personnel in the workforce (Griffiths et al. 2019).

Although the figures from SANC indicates that there is a sufficient number of nurses to deliver healthcare services in South Africa, several sources of information show that the country has a shortage of nurses, which will persist into the future as there are fewer enrolments for the nursing qualification (FIND 2020; Mbombi & Mothiba 2020; Van Rensburg 2019).

The skill level of the South African nursing workforce is low, as 45% of nurses are not qualified registered nurses, which is not reflected in the nurses-to-population ratio (SANC 2021). The low number of registered nurses ultimately leads to increased harm to patients and a deterioration of the quality of care provided within the healthcare system.

It is concerning that close to 40% of the South African nursing workforce is due for retirement during the next 10 to 15 years (SANC 2021). This, combined with a reduced pipeline of newly qualified nurses, will result in additional severe shortages of skilled nurses and poor health outcomes for the South African nation (Mutshatshi et al. 2018). South African Nursing Council can primarily be held responsible for the decrease in nursing student outputs because of their sluggish implementation of a new syllabus for nurses since 2012 and the lack of accreditation of private nursing institutions, which have historically trained 60% of all nurses in the country (Mutshatshi et al. 2018).

South Africa’s healthcare sector is fragmented causing further serious deficits in healthcare workers because of the uneven distribution of human resources for health in the country, which is mostly felt at the nursing level as they are at the forefront of healthcare service delivery (Van Rensburg 2014, as cited by Maphumulo & Bhengu 2019). Although most of the citizens depend on the public healthcare for services, it is under-resourced when compared to private healthcare (WHO 2015; Van Rensburg 2014, as cited by Maphumulo & Bhengu 2019). Other healthcare worker shortages in South Africa emanate from an inequitable distribution of workers between urban and rural areas and affluent versus poorer provinces within the public healthcare sector (HR for Health SA 2030 2011). This creates a situation where privileged South African citizens who are in the minority have access to optimal healthcare services while the poor receive substandard healthcare. Although the density of healthcare workers in the private healthcare sector is higher than in the public sector, both sectors are experiencing difficulties in filling their vacancies, which indicates that there are not enough nurses to fill these positions (Pretorius & Klopper 2012 as cited in Maphumulo & Bhengu 2019).

The South African government intends to roll out the National Health Insurance (NHI) as a panacea to solve the fragmentation of healthcare services and to provide equitable healthcare to all South African citizens (HR for Health SA 2030 2011). The findings of this research project indicate that South Africa is unable to execute the NHI based on the proposed system, requiring significantly more skilled healthcare workers to operate effectively.

### Specific objective: To describe the attrition rate of healthcare professionals in South Africa

The NDOH recorded a turnover rate of 14% for professional nurses in its 2018/2019 Annual Report (NDOH 2019). Provincial health departments reported turnover rates for professional nurses of 10% for both the Eastern Cape and Gauteng for the year 2018/2019 (EDOH 2019; Gauteng Department of Health (GDOH) 2019). This turnover rate indicates that the public healthcare sector has to replace 14% of its most qualified nurses annually.

The South African government estimates that 25% of healthcare professionals are lost to migration annually, but unfortunately, there are no reliable sources of information concerning the emigration of these professionals, and hence, this figure could not be verified through this research study (NDOH 2011). Furthermore, the various Departments of Health do not appear to record turnover statistics consistently. The reasons why workers leave are not recorded, and therefore, it is not known where these workers are lost to. Numerous studies have been conducted concerning the brain drain of healthcare workers in South Africa, and these studies have provided different estimations of emigration by these workers as well as given diverse reasons for the occurrence of this situation in the country (WHO 2015). The migration of nurses within the healthcare sector, as well as emigration to other countries, has been cited as two significant factors that have added to the high attrition rate of professional nurses in the country (WHO 2015). Although there have been a plethora of studies supporting the occurrence of a brain drain of healthcare workers from South Africa, the research conducted through this study could not confirm whether this phenomenon exists among the country’s professional nurses. This is mainly as a result of a lack of tracking by the South African government and other healthcare bodies of the movement of healthcare workers in and out of the country. A study conducted by the WHO in 2017 confirms that proof of extensive migration by South African nurses is hard to verify empirically, despite several past research studies being conducted on this topic (WHO 2017).

The South African Nursing Council produces a report for the number of nurses who have requested that verifications of their training transcripts or qualifications be sent to other countries, which is known as the Verification and Transcript Statistics report (SANC 2020). The WHO 2017 study uses this report as an indication that South Africa does not appear to be experiencing a brain drain of nurses based on the low number of requests for verifications by nurses. Furthermore, the study indicates that over 5 years, only 2158 professional nurses requested verification letters from SANC, either personally or via recruitment agencies (WHO 2017). The Verification and Transcript Statistics report is unfortunately not a good way to determine whether or not South Africa is experiencing a brain drain of professional nurses as it specifically states that the figures provided are merely an indication of the number of nurses who have made requests for these documents to be sent to the various countries specified on the report. When a nurse has requested a verification, it does not automatically mean that he or she has taken up employment in these countries, and hence, this report cannot confirm the extent of emigration of South African nurses (SANC 2020). Furthermore, nurses are not obligated to inform SANC that they have left the country (SANC 2020).

### Recommendations

These recommendations are aimed at improving the effectiveness of healthcare delivery, ensuring equitable access to care, and enhancing the sustainability of the nursing profession in the country.

Firstly, South Africa should adopt the WHO’s National Health Workforce Accounts (NHWA) framework. The NHWA provides a standardised approach to collecting, analysing and using health workforce data across various dimensions, including active practitioner status, skills mix and migration patterns. Implementing this system would significantly improve the country’s ability to track the internal and external movements of nurses and address current limitations in workforce planning, where figures are often based on outdated or aggregated registration data. Accurate and disaggregated data would enable policymakers to develop responsive strategies to address staffing gaps and predict future workforce needs more effectively.

Secondly, there is a pressing need to revise the current approach to workforce planning. Traditional reliance on nurse-to-population ratios does not capture the complexity of healthcare needs or the functionality of the nursing workforce. Therefore, South Africa should transition from purely numerical ratios to a competency- and service-delivery-based planning model. This revised model should consider the clinical competencies required to address the country’s burden of disease, such as HIV/AIDS and tuberculosis, the level of care complexity in various regions, and the actual service coverage provided by nurses in different contexts. By focusing on the availability of appropriately skilled nurses rather than headcount alone, the healthcare system can be more responsive to population health needs.

Furthermore, investment in nursing education and training is another crucial area that requires immediate attention. There is an urgent need to expand the capacity of nursing colleges and accredited training institutions to produce a sufficient number of qualified nurses. This expansion must be accompanied by increased financial support for students, especially those from disadvantaged backgrounds, to reduce attrition rates in nursing programmes. Moreover, improvements in the quality and accessibility of clinical placements and mentorship during training can enhance practical competence and readiness for service. Strengthening partnerships between the Department of Health and higher education institutions is also essential to ensure that nursing graduates meet the evolving demands of healthcare delivery.

Thirdly, the development and implementation of comprehensive retention strategies are critical. The high attrition rates among professional nurses, particularly in the public sector, are driven by poor working conditions, low remuneration, limited career advancement and inadequate support systems. To reverse this trend, healthcare institutions must improve remuneration packages to reflect the demanding nature of nursing work. In addition, enhancing workplace safety, resource availability and professional recognition can contribute significantly to job satisfaction and morale. Institutions should also implement mechanisms such as regular staff feedback, performance recognition and wellness programmes to support nurses’ mental and emotional well-being. Conducting exit interviews and workforce surveys can further help identify root causes of turnover and inform proactive management responses.

**FIGURE 6:**
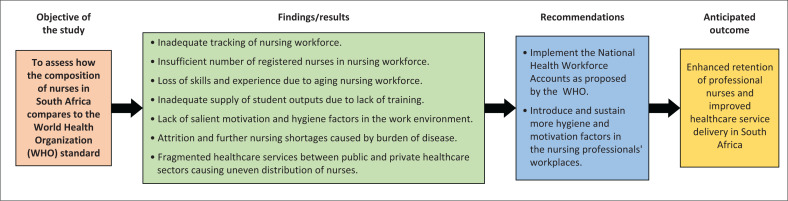
Recommendations based on the results of the study.

The recommendations, based on the findings of the study, are presented in Figure 6, created by the authors, which, when effectively implemented, have the potential to enhance the retention of professional nurses, thereby contributing to improved healthcare service delivery in South Africa.

## Conclusion

South Africa’s nurses-to-population ratio is above the WHO standard, but it is not an accurate portrayal of the nursing sector in the country because of several factors that have been identified as negatively impacting the figures. These factors include the lack of reliable data for the nursing workforce, the varying skill level of nurses, the age distribution of nurses, the burden of disease in the country and the uneven distribution of healthcare workers between the public and private healthcare sectors. The SANC is the only organisation in the country that collects workforce data for nurses, but the statistics for nurses are unreliable because of poor data collection methods, which do not track the whereabouts of nurses (Brits 2019; WHO 2017). Although the figures from SANC indicates that there is a sufficient number of nurses to deliver healthcare services in South Africa, several sources of information show that the country has a shortage of nurses which will persist into the future as there are fewer enrolments for the nursing qualification (FIND 2020; Van Diemen 2019; Van Rensburg 2019).

As nurses are essential for the delivery of healthcare services, the impact of the nursing shortage in the country can adversely affect the general health of the South African population, as more nurses per unit of population are associated with a healthier nation (Bigbee 2009; NDOH 2011). The shortage of nurses in South Africa also results in a low nurse-patient ratio as nurses have to care for a higher number of patients who directly affects service delivery, that is, the quality of healthcare received in South African hospitals as sub-optimal nurse staffing is linked to a risk of adverse incidents and patient deaths (DENOSA 2012; Fagerström et al. 2018).

The South African public healthcare sector loses 14% of its nursing workforce to attrition annually, based on the annual report by the NDOH (2019). Workforce data for nurses are collected inconsistently across the various provincial health departments in the country, and therefore, it is not known whether this figure is correct. Furthermore, the government does not collect information about the reasons why nurses are leaving the public sector and hence is unable to develop and implement suitable retention strategies aimed at improving the retention of nurses, which is crucial in a situation where the country is experiencing a shortage of this critical skill.
